# Association Between Age at Diagnosis of Type 2 Diabetes and Cardiovascular Diseases: A Nationwide, Population-Based, Cohort Study

**DOI:** 10.3389/fendo.2021.717069

**Published:** 2021-10-04

**Authors:** Chunyan Hu, Lin Lin, Yujing Zhu, Yi Zhang, Shuangyuan Wang, Jie Zhang, Hongyan Qi, Mian Li, Yuanyue Zhu, Yanan Huo, Qin Wan, Yingfen Qin, Ruying Hu, Lixin Shi, Qing Su, Xuefeng Yu, Li Yan, Guijun Qin, Xulei Tang, Gang Chen, Min Xu, Yu Xu, Tiange Wang, Zhiyun Zhao, Zhengnan Gao, Guixia Wang, Feixia Shen, Zuojie Luo, Li Chen, Qiang Li, Zhen Ye, Yinfei Zhang, Chao Liu, Youmin Wang, Tao Yang, Huacong Deng, Lulu Chen, Tianshu Zeng, Donghui Li, Jiajun Zhao, Yiming Mu, Yufang Bi, Weiqing Wang, Guang Ning, Shengli Wu, Yuhong Chen, Jieli Lu

**Affiliations:** ^1^ Department of Endocrine and Metabolic Diseases, Shanghai Institute of Endocrine and Metabolic Diseases, Ruijin Hospital, Shanghai Jiao Tong University School of Medicine, Shanghai, China; ^2^ Shanghai National Clinical Research Center for Metabolic Diseases, Key Laboratory for Endocrine and Metabolic Diseases of the National Health Commission of the PR China, Shanghai National Center for Translational Medicine, Ruijin Hospital, Shanghai Jiao Tong University School of Medicine, Shanghai, China; ^3^ Department of Endocrinology, Karamay Municipal People’s Hospita , Xinjiang, China; ^4^ Jiangxi Provincial People’s Hospital, Affiliated to Nanchang University, Nanchang, Xinjiang, China; ^5^ Department of Endocrinology, The Affiliated Hospital of Luzhou Medical College, Luzhou, China; ^6^ Department of Endocrinology, The First Affiliated Hospital of Guangxi Medical University, Nanning, China; ^7^ Zhejiang Provincial Center for Disease Control and Prevention, Hangzhou, China; ^8^ Department of Endocrinology, Affiliated Hospital of Guiyang Medical University, Guiyang, China; ^9^ Xinhua Hospital, Affiliated to Shanghai Jiaotong University School of Medicine, Shanghai, China; ^10^ Tongji Hospital, Tongji Medical College, Huazhong University of Science and Technology, Wuhan, China; ^11^ Sun Yat-sen Memorial Hospital, Sun Yat-sen University, Guangzhou, China; ^12^ Department of Endocrinology, The First Affiliated Hospital of Zhengzhou University, Zhengzhou, China; ^13^ Department of Endocrinology, The First Hospital of Lanzhou University, Lanzhou, China; ^14^ Fujian Provincial Hospital, Fujian Medical University, Fuzhou, China; ^15^ Dalian Municipal Central Hospital, Affiliated of Dalian Medical University, Dalian, China; ^16^ Department of Endocrinology, The First Hospital of Jilin University, Changchun, China; ^17^ Department of Endocrinology, The First Affiliated Hospital of Wenzhou Medical University, Wenzhou, China; ^18^ Qilu Hospital of Shandong University, Jinan, China; ^19^ Department of Endocrinology, The Second Affiliated Hospital of Harbin Medical University, Harbin, China; ^20^ Department of Endocrinology, Central Hospital of Shanghai Jiading District, Shanghai, China; ^21^ Department of Endocrinology, Jiangsu Province Hospital on Integration of Chinese and Western Medicine, Nanjing, China; ^22^ Department of Endocrinology, The First Affiliated Hospital of Anhui Medical University, Hefei, China; ^23^ Department of Endocrinology, The First Affiliated Hospital of Nanjing Medical University, Nanjing, China; ^24^ Department of Endocrinology, The First Affiliated Hospital of Chongqing Medical University, Chongqing, China; ^25^ Union Hospital, Tongji Medical College, Huazhong University of Science and Technology, Wuhan, China; ^26^ Department of Gastrointestinal Medical Oncology, the University of Texas MD Anderson Cancer Center, Houston, TX, United States; ^27^ Shandong Provincial Hospital, Affiliated to Shandong University, Jinan, China; ^28^ Department of Endocrinology, Chinese People’s Liberation Army General Hospital, Beijing, China

**Keywords:** cardiovascular disease, type 2 diabetes, early-onset diabetes, age at diagnosis of diabetes, obesity

## Abstract

**Objectives:**

Nationwide studies focusing on the impact of early-onset type 2 diabetes and obesity on the development of cardiovascular diseases (CVD) are limited in China. We aimed to investigate the association between age at diagnosis of type 2 diabetes and the risk of CVD, and to further examine the modifying effect of obesity on this association among Chinese adults.

**Methods:**

This study included 23,961 participants with previously diagnosed diabetes from a large nationwide population-based cohort study across mainland China. With an interviewer-assisted questionnaire, we collected detailed information on CVDs. Logistic regression analysis was used to evaluate the risk of CVDs associated with age at diagnosis of diabetes.

**Results:**

Compared with patients with late-onset diabetes (≥60 years), those with earlier-onset diabetes had increased risks for CVD, with adjusted ORs (95% CIs) of 1.72 (1.36-2.17), 1.52 (1.31-1.75) and 1.33 (1.19-1.48) for patients diagnosed aged <40, 40-49 and 50-59 years, respectively. Each 5-year earlier age at diagnosis of type 2 diabetes was significantly associated with 14% increased risk of CVD (OR, 1.14; 95%CI, 1.11-1.18). This association was more prominent for patients with obesity than those with normal body mass index (BMI). Significant interaction was detected between age at diagnosis and BMI categories on CVD risk (*P* for interaction=0.0457).

**Conclusion:**

Early-onset type 2 diabetes was significantly associated with higher risk of CVD, and this association was more prominent among patients with obesity.

## Introduction

Type 2 diabetes has become a major health burden worldwide. In the past few decades, the prevalence of diabetes in China has increased dramatically, with 12.8% of adults of diabetes and 35.2% of prediabetes in 2017 (1, 2). Although type 2 diabetes was conventionally recognized as a disease of the middle-aged and elderly, a rapid growth of type 2 diabetes has been observed among younger adults and even adolescents (3, 4), especially in developing countries. In Asia, one in five patients with diabetes was diagnosed before 40 years (5). Early-onset type 2 diabetes was reported to be associated with poor metabolic control and accelerated development of complications (5–12). Although the impact of age at diagnosis of type 2 diabetes on the risk of cardiovascular disease (CVD) has been investigated in previous studies (8, 9, 13–17), the results remain inconclusive. Some studies proposed inverse association between age at diagnosis of diabetes and risk of CVD (8, 14, 15), whereas others suggested positive (16, 17) or null association (9, 18) in prospective analyses.

During the past decades, China has experienced a rapid transition to western lifestyles with more sedentary behavior and a high energy/fat diet (19), leading to the escalating rates of obesity and related metabolic diseases, including type 2 diabetes. The rise in the prevalence of obesity in early and middle-aged population is likely to increase the incidence of early-onset type 2 diabetes (20). Previous studies have reported a strong relationship between obesity in youth and subsequent onset of type 2 diabetes (20–23). Obesity was also an independent risk factor for CVD, which is the leading cause of premature death in China (24). However, to the best of our knowledge, studies investigating the effect of obesity on the association between age at diagnosis of type 2 diabetes and the risk of CVD are limited.

Therefore, using the comprehensive data from the Risk Evaluation of cAncers in Chinese diabeTic Individuals: a lONgitudinal (REACTION) study, we aim to investigate the association between age at diagnosis of type 2 diabetes and the risk of CVDs, and to further evaluate the modifying effect of BMI on this association among Chinese adults.

## Materials and Methods

### Study Population

The study population was derived from the REACTION study, including 259,657 participants from 25 communities across mainland China. The details of the study population were described previously (25, 26). Briefly, community dwelling adults aged 40 years or older were invited to participate the baseline survey during 2011-2012. There was no restriction on sex or ethnicity. Participants who signed informed consents were recruit and scheduled for a comprehensive clinical examination (including physical and biochemical measurements) and a structured questionnaire interview. Among 259,657 participants in the REACTION study, 26,795 individuals reported having ever been diagnosed as type 2 diabetes by professional physicians. We further excluded participants with missing information about the time of diagnosis (n=2797) or those diagnosed before 18 years (n=37). Finally, 23,961 individuals were included in the current analysis.

The REACTION study was approved by the ethic committee of Ruijin hospital affiliated to Shanghai Jiaotong University School of Medicine. All participants provided written informed consent.

### Data Collection

During the personal interview, information on sociodemographic characteristics, lifestyle factors (including smoking or drinking status, physical activity, and dietary habits), and medical history was collected using a standard questionnaire. Education levels were divided into less than high school versus high school education or above. Current smoking was defined as smoking cigarettes one per day or seven per week regularly during the past 6 months. People who consumed alcohol once per week regularly during the past 6 months was considered current drinker. Physical activity was estimated using the short form of the International Physical Activity Questionnaire. Participants were asked about the questions on the intensity, duration, and frequency of physical activity at leisure time. According to this information, total metabolic equivalents were calculated to measure the physical activity levels. Moderate and vigorous physical activity refers to moderate intensity activity ≥ 150 min/wk, or vigorous intensity or combination activity ≥ 75 min/wk. A dietary questionnaire was used to collect the information on the frequency and quantity of major food items (such as red meat, fruits and vegetables, dairy, soy, and Chinese traditional food) over the past 12 months. According to the recommendation of the American Heart Association (27), a healthy diet was defined as a diet score ≥ 3, including the following 4 components: fruits and vegetables ≥ 4.5 cups/d, fish ≥ 198 g/wk, sweets/sugar-sweetened beverages ≤ 450 kcal/wk, and soy protein ≥ 25 g/d. Probable depression was accessed using the patient health questionnaire (PHQ)–9 with the total score of ≥10 (28).

All participants underwent measurements on height, weight, and blood pressure by experienced nurses. Height and weight were measured using standard protocol with participants wearing lightweight clothes and no shoes. BMI was calculated as weight in kilograms divided by height in meters squared (kg/m^2^). Blood pressure was tested three times consecutively at 1-min intervals with an automated electronic device (OMRON Model HEM-725 FUZZY, Omron Company, Dalian, China). The average of the three readings for systolic or diastolic blood pressure was used to analyze.

After 10 hours of overnight fasting, a blood sample was collected and aliquoted into 0.5-mL Eppendorf tubes within 2 hours. Sera was then shipped by air in dry ice to the central laboratory of the study located at Shanghai Institute of Endocrine and Metabolic Diseases, which is certificated by the U.S. National Glycohemoglobin Standardization Program and passed the Laboratory Accreditation Program of the College of American Pathologists. The level of HbA1c was determined by the method of high-performance liquid chromatography (VARIANT™ II and D-10™ Systems, BIO-RAD, Hercules, CA, USA) in the central laboratory. Total cholesterol and triglycerides were measured with an autoanalyzer (ARCHITECT c16000 System, Abbott Laboratories, IL, USA).

### Classification and Definition

The date of diagnosis was recorded for each participant who reported having type 2 diabetes. Age at diagnosis of type 2 diabetes was categorized as < 40 years, 40-49 years, 50-59 years and ≥ 60 years. Obesity was defined as a BMI of ≥ 28.0 kg/m^2^, and overweight was defined as a BMI of 24.0-27.9 kg/m^2^.

We collected information on CVDs with an interviewer-assisted questionnaire. An open-ended question was asked: “Has a doctor or other health professional ever told you that you have myocardial infarction, coronary heart disease (CHD), or stroke?” Total CVD in the analysis referred to the composite of the three CVDs (reported myocardial infarction, CHD, or stroke). Validation of the self-reported CVDs from questionnaire was performed in Shanghai Youyi Community, 1 of the 25 communities in the REACTION study. Two physicians who were blind to the self-reported data reviewed the related hospitalization records, and classified the cases as definite, questionable, or misdiagnosed. The validation rate of CVDs was 91.1% (29, 30).

### Statistical Analysis

Continuous variables are presented as means ± SDs or medians (IQRs) and categorical variables are presented as number (proportions). The *P*_trend_ for general characteristics according to age at diagnosis of diabetes was calculated using linear regression analyses for continuous variables and logistic regression for categorical variables. Multivariable adjusted logistic regression analyses were performed to estimate the association of age at diagnosis of type 2 diabetes on the risk of CVDs, with the group of the latest-onset type 2 diabetes (≥60 years) as the reference. Model 1 was adjusted for age and sex; Model 2 was adjusted for age, sex, education, smoking status, drinking status, physical activity, healthy dietary, BMI; Model 3 was further adjusted for systolic blood pressure, total cholesterol, triglycerides and HbA1c. Age at diagnosis of type 2 diabetes was also analyzed as continuous variables and the association between every 5-year earlier age at diabetes diagnosis and CVD risk was estimated.

Stratified analysis was conducted according to BMI categories. To explore the possible interaction between age at diagnosis of diabetes and BMI in the development of CVDs, we used the cross-products of age at diagnosis of diabetes and BMI categories as the interaction terms. The likelihood ratio test was used to analyze the potential interaction by comparing the full model including the interaction item with the simplified model without the interaction item. We further examined the combined effects of BMI and age at diagnosis of diabetes on the risk of CVDs.

All analyses were conducted using SAS 9.4 (SAS Institute, Cary, NC). A two-tailed p<0.05 was considered statistically significant.

## Results

### Characteristics of the Study Participants

This study included 23,961 people with previously diagnosed type 2 diabetes. Overall, the mean (± SD) age at diagnosis of diabetes was 54.60 ± 9.48 years. A total of 3653 (15.25%) participants have reported CVD in this study. Characteristics of the study population according to age at diagnosis of type 2 diabetes were presented in **(Table 1)**. On average, patients with earlier age at diagnosis were much younger, and they are more likely to have poor glycemic control, probable depression, and high triglycerides, but lower BMI and systolic blood pressure. Besides, Individuals with earlier age at diagnosis of diabetes have higher percentage of insulin-user, while lipid-lowering treatment and antihypertensive drugs are less used (all *P* for trend < 0.05, **Table 1**).

**Table 1:** Baseline characteristics according to age at diagnosis of diabetes.

	Age at diagnosis of diabetes (years)				
	18-39	40-49	50-59	≥60	*P*_trend_
No. of participants (%)	1413 (5.90)	6103 (25.47)	9827 (41.01)	6618 (27.62)	
Age at baseline, years	50.81 ± 8.43	54.82 ± 7.02	61.46 ± 5.83	70.59 ± 5.76	<.0001
Male gender, n (%)	669 (47.35)	2653 (43.47)	3814 (38.81)	2593 (39.18)	<.0001
BMI, kg/m^2^	25.28 ± 3.66	25.43 ± 3.65	25.53 ± 3.62	25.60 ± 3.60	0.0006
BMI < 24kg/m^2^, n(%)	573 (40.55)	2225 (36.46)	3469 (35.30)	2240 (33.85)	<.0001
BMI 24-27.9 kg/m^2^, n(%)	559 (39.56)	2693 (44.13)	4299 (43.75)	2942 (44.45)	0.0325
BMI ≥28 kg/m^2^, n(%)	281 (19.89)	1185 (19.42)	2059 (20.95)	1436 (21.70)	0.0022
High school or above education, n (%)	693 (49.04)	2644 (43.32)	3411 (34.71)	2109 (31.87)	<.0001
Current cigarette smoking, n (%)	291 (20.59)	1083 (17.75)	1273 (12.95)	576 (8.70)	<.0001
Current alcohol drinking, n (%)	143 (10.12)	578 (9.47)	732 (7.45)	379 (5.73)	<.0001
Moderate and vigorous physical activity, n (%)	181 (12.81)	831 (13.62)	1387 (14.11)	877 (13.25)	0.4462
Healthy diet, n (%)	171 (14.12)	785 (14.69)	1179 (13.84)	700 (12.22)	0.0005
Triglycerides, mmol/L	2.02 ± 1.81	2.01 ± 1.69	1.92 ± 1.47	1.80 ± 1.23	<.0001
Total cholesterol, mmol/L	4.95 ± 1.23	4.93 ± 1.18	4.95 ± 1.19	4.90 ± 1.18	0.1024
Systolic blood pressure, mmHg	132.98 ± 20.22	135.42 ± 20.24	139.77 ± 20.47	144.42 ± 20.65	<.0001
HbA1c, %	7.80 (6.80-9.40)	7.40 (6.60-8.80)	7.10 (6.40-8.10)	6.90 (6.30-7.80)	<.0001
Glucose-lowering treatment, n (%)	1169 (82.73)	4882 (79.99)	7762 (78.99)	5077 (76.72)	<.0001
Insulin therapy, n (%)	561 (39.70)	1632 (26.74)	1784 (18.15)	820 (12.39)	<.0001
Oral hypoglycemic drugs use, n (%)	861 (60.93)	4068 (66.66)	6877 (69.98)	4677 (70.67)	<.0001
Lipid-lowering treatment, n (%)	18 (1.27)	138 (2.26)	239 (2.43)	170 (2.57)	0.0136
Antihypertensive drugs use, n (%)	230 (16.28)	1220 (19.99)	2523 (25.67)	2234 (33.76)	<.0001
Probable depression, n (%)	34 (2.41)	119 (1.95)	151 (1.54)	95 (1.44)	0.0019

### Association of Age at Diagnosis of Diabetes With CVD

The association between age at diagnosis of type 2 diabetes and the risk of CVD is presented in **Table 2**. In age and sex-adjusted model, compared with late-onset diabetes (diagnosed ≥60 years), young-onset diabetes (diagnosed <40 years of age) was associated with 46%, 56%, 77%, and 38% increased risk for total CVD, stroke, myocardial infarction, and CHD, respectively. Further adjustment for lifestyle factors and metabolic measurements did not change the estimates significantly. Compared with patients diagnosed ≥60 years, the fully adjusted ORs (95%CIs) of total CVD for patients with adjusted ORs (95% CIs) of 1.72 (1.36-2.17), 1.52 (1.31-1.75) and 1.33 (1.19-1.48) for patients diagnosed aged <40, 40-49 and 50-59 years, respectively. Each 5-year younger age at diagnosis of type 2 diabetes was significantly associated with an increased risk of CVD (OR, 1.14; 95%CI, 1.11-1.18). Results of individual CVD components were similar. Earlier age at diagnosis of diabetes was also strongly associated with stroke, myocardial infarction, and CHD, with the ORs (95%CIs) for per 5-year earlier of age at diagnosis of type 2 diabetes were 1.11 (1.06-1.17), 1.17 (1.08-1.26), and 1.13 (1.10-1.17), respectively, in fully adjusted analyses. We did not observe sex differences for the association between age at diagnosis of diabetes and risk of CVD (*P* for interaction >0.05, **Supplementary Table 1**).

**Table 2:** Odds ratio (95% CI) for cardiovascular disease risks according to age at diagnosis of diabetes.

	Age at diagnosis of diabetes (years)				
	≥60	50-59	40-49	18-39	Per 5-years earlier
**Cardiovascular disease, case (%)**	1337 (20.20)	1475 (15.01)	692 (11.34)	149 (10.54)	
Model 1	1.00 (reference)	1.21 (1.10-1.33)	1.28 (1.13-1.46)	1.46 (1.19-1.79)	1.09 (1.07-1.12)
Model 2	1.00 (reference)	1.31 (1.18-1.46)	1.45 (1.26-1.67)	1.61 (1.28-2.02)	1.12 (1.09-1.16)
Model 3	1.00 (reference)	1.33 (1.19-1.48)	1.52 (1.31-1.75)	1.72 (1.36-2.17)	1.14 (1.11-1.18)
**Stroke, case (%)**	350 (5.29)	365 (3.71)	174 (2.85)	37 (2.62)	
Model 1	1.00 (reference)	1.24 (1.04-1.47)	1.39 (1.11-1.76)	1.56 (1.07-2.28)	1.09 (1.04-1.14)
Model 2	1.00 (reference)	1.26 (1.05-1.52)	1.42 (1.11-1.82)	1.50 (0.99-2.28)	1.10 (1.05-1.15)
Model 3	1.00 (reference)	1.26 (1.05-1.52)	1.47 (1.14-1.89)	1.61 (1.05-2.45)	1.11 (1.06-1.17)
**Myocardial infarction, case (%)**	124 (1.87)	137 (1.39)	84 (1.38)	20 (1.42)	
Model 1	1.00 (reference)	1.15 (0.88-1.52)	1.49 (1.05-2.11)	1.77 (1.03-3.02)	1.13 (1.06-1.21)
Model 2	1.00 (reference)	1.26 (0.94-1.71)	1.59 (1.09-2.32)	2.22 (1.26-3.91)	1.18 (1.09-1.27)
Model 3	1.00 (reference)	1.25 (0.92-1.69)	1.52 (1.03-2.24)	2.14 (1.21-3.79)	1.17 (1.08-1.26)
**Coronary heart disease, case (%)**	1021 (15.43)	1142 (11.62)	525 (8.60)	113 (8.00)	
Model 1	1.00 (reference)	1.20 (1.07-1.33)	1.23 (1.06-1.41)	1.38 (1.09-1.74)	1.08 (1.05-1.11)
Model 2	1.00 (reference)	1.32 (1.17-1.48)	1.43 (1.22-1.67)	1.54 (1.19-2.00)	1.12 (1.08-1.15)
Model 3	1.00 (reference)	1.35 (1.20-1.52)	1.49 (1.27-1.75)	1.65 (1.27-2.15)	1.13 (1.10-1.17)

Model 1, adjusted for age and sex; Model 2: adjusted for age, sex, education, smoking status, drinking status, physical activity, healthy dietary, body mass index; Model 3, further adjusted for systolic blood pressure, total cholesterol, triglycerides and HbA1c.

Sensitivity analysis was performed to evaluate the modification of the medication use on the association. After further adjustment for insulin therapy and oral hypoglycemic drugs, the estimates of CVD risk moderated but remained significant, with the OR (95%CI) of 1.25 (1.09-1.79), 1.32 (1.13-1.54), 1.39 (1.09-1.79) for individuals diagnosed at 50-59, 40-49 and 18-39 years, respectively, compared with participants diagnosed ≥60 years. Each 5-year younger age at diagnosis of diabetes was significantly associated with 10% increased risk of CVD (OR, 1.10; 95%CI, 1.17-1.14). Further adjustment for lipid-lowering treatment, antihypertensive drugs use and probable depression, the estimates did not change significantly (**Supplementary Table 2**).

### The Interaction Effect of BMI on the Association of Age at Diagnosis of Diabetes With CVD

In the BMI stratified analysis, earlier age at diagnosis of type 2 diabetes was associated with higher risk of total CVD, and the association was more pronounced among participants with obesity (**Table 3**). After multivariable adjustment, every 5 years earlier of age at diagnosis of diabetes was significantly associated with the ORs (95%CIs) of CVD of 1.09 (1.03-1.15), 1.14 (1.09-1.19), and 1.24 (1.17-1.33) among participants with normal weight, overweight, and obesity, respectively. Significant interaction was found between age at diagnosis of diabetes and BMI categories for risk of total CVD (*P* for interaction= 0.0457). Significant increased risk of stroke was observed for those with earlier age at diagnosis of type 2 diabetes among people with overweight or obesity, while no significant association was observed among patients with normal weight (*P* for interaction= 0.0205). The association between age at diagnosis of diabetes and the risk of myocardial infarction or CHD was directionally consistent with the combined analysis of total CVD, although the interactions were not significant (*P* for interaction = 0.1931 for myocardial infarction, and *P* for interaction = 0.1568 for CHD).

**Table 3:** Association between age at diagnosis of diabetes and cardiovascular disease risk by BMI categories.

	Cardiovascular disease		Stroke		Myocardial infarction		Coronary heart disease	
	Case (%)	OR (95%CI)	Case (%)	OR (95%CI)	Case (%)	OR (95%CI)	Case (%)	OR (95%CI)
**BMI< 24kg/m^2^**								
≥60	366 (16.34)	1.00 (reference)	107 (4.78)	1.00 (reference)	36 (1.61)	1.00 (reference)	270 (12.05)	1.00 (reference)
50-59	399 (11.50)	1.29 (1.06-1.57)	107 (3.08)	1.02 (0.73-1.41)	27 (0.78)	0.83 (0.46-1.51)	304 (8.76)	1.39 (1.11-1.74)
40-49	175 (7.87)	1.30 (1.002-1.69)	47 (2.11)	1.09 (0.70-1.70)	17 (0.76)	0.99 (0.46-2.13)	128 (5.75)	1.29 (0.96-1.75)
18-39	49 (8.55)	1.81 (1.21-2.69)	10 (1.75)	1.01 (0.46-2.23)	5 (0.87)	1.70 (0.58-4.98)	38 (6.63)	1.89 (1.20-2.96)
Per 5-years earlier		1.09 (1.03-1.15)		1.01 (0.92-1.10)		1.09 (0.95-1.26)		1.10 (1.03-1.16)
**BMI 24-27.9 kg/m^2^**								
≥60	614 (20.87)	1.00 (reference)	163 (5.54)	1.00 (reference)	59 (2.01)	1.00 (reference)	462 (15.70)	1.00 (reference)
50-59	684 (15.91)	1.34 (1.15-1.56)	167 (3.88)	1.37 (1.04-1.80)	74 (1.72)	1.33 (0.86-2.06)	530 (12.33)	1.35 (1.14-1.61)
40-49	325 (12.07)	1.49 (1.21-1.84)	86 (3.19)	1.63 (1.13-2.35)	46 (1.71)	1.66 (0.95-2.90)	237 (8.80)	1.39 (1.10-1.76)
18-39	59 (10.55)	1.53 (1.07-2.19)	16 (2.86)	1.90 (1.02-3.54)	8 (1.43)	2.01 (0.84-4.81)	46 (8.23)	1.49 (1.003-2.23)
Per 5-years earlier		1.14 (1.09-1.19)		1.13 (1.05-1.22)		1.18 (1.06-1.32)		1.13 (1.07-1.18)
**BMI ≥ 28kg/m^2^**								
≥60	357 (24.86)	1.00 (reference)	80 (5.57)	1.00 (reference)	29 (2.02)	1.00 (reference)	289 (20.13)	1.00 (reference)
50-59	392 (19.04)	1.44 (1.16-1.79)	91 (4.42)	1.45 (0.97-2.16)	36 (1.75)	1.74 (0.94-3.24)	308 (14.96)	1.40 (1.11-1.77)
40-49	192 (16.20)	1.99 (1.48-2.68)	41 (3.46)	1.78 (1.03-3.08)	21 (1.77)	2.13 (0.95-4.81)	160 (13.50)	2.12 (1.54-2.91)
18-39	41 (14.59)	2.30 (1.45-3.67)	11 (3.91)	2.30 (1.003-5.29)	7 (2.49)	3.33 (1.10-10.10)	29 (10.32)	1.96 (1.16-3.31)
Per 5-years earlier		1.24 (1.17-1.33)		1.24 (1.12-1.38)		1.25 (1.07-1.45)		1.22 (1.14-1.30)

Adjusted for age, sex, education, smoking status, drinking status, physical activity, healthy dietary, systolic blood pressure, total cholesterol, triglycerides and HbA1c. P for interaction for cardiovascular disease = 0.0457; p for interaction for stroke = 0.0205; p for interaction for myocardial infarction= 0.1931; p for interaction for coronary heart disease = 0.1568. BMI, body mass index.

**Figure 1:**
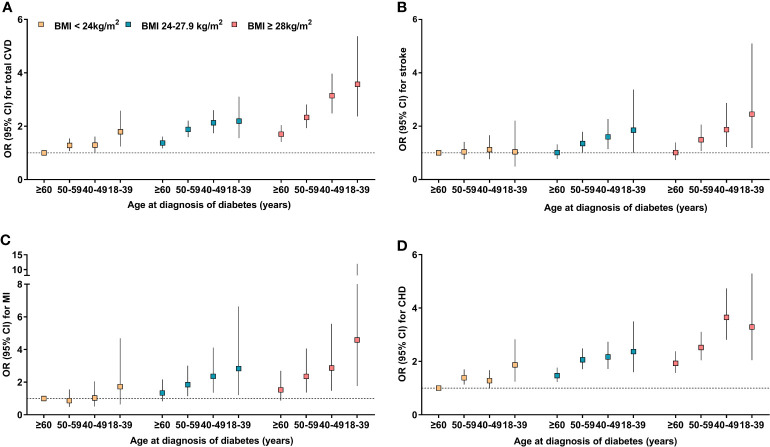
Adjusted odds ratios for cardiovascular disease risk according to combinations of BMI and age at diagnosis of diabetes. **(A)** cardiovascular disease; **(B)** Stroke; **(C)** Myocardial infarction; **(D)** Coronary heart disease. ORs (95% CIs) were adjusted for age, sex, education, smoking status, drinking status, physical activity, healthy dietary, systolic blood pressure, total cholesterol, triglycerides and HbA1c.

We further explored the combined effects of BMI and age at diagnosis of diabetes on the risk of CVD (**Figure 1**). Multivariate-adjusted logistic regression analysis revealed that compared with the reference group (who were diagnosed ≥60 years and with normal weight), those with earlier age at diagnosis and with obesity or overweight had significantly increased risk of total CVD or individual CVD components. Overall, the ORs (95%CI) for risk of total CVD, stroke, myocardial infarction, and CHD associated early age at diagnosis of diabetes and obesity (diagnosed <40 years and with obesity *vs* diagnosed ≥60 years and with normal weight) were 3.57 (2.37-5.37), 2.45 (1.18-5.10), 4.58 (1.76-11.96), and 3.29 (2.05-5.29), respectively.

## Discussion

In this large population-based study, we found that age at diagnosis of type 2 diabetes was associated with the risk of total CVD, stroke, myocardial infarction, and CHD among Chinese adults. The association of age at diagnosis of type 2 diabetes with risk of CVD was more pronounced among patients with obesity compared to those with normal weight, indicating that obesity might modify the association between age at diagnosis of type 2 diabetes and CVD risk. To the best of our knowledge, this is the first nation-wide cohort study to explore the modifying effect of obesity on the association between diabetes onset age and the CVD risk. These findings have important clinical implications.

Young-onset type 2 diabetes has been associated with an increased CVD risk in previous epidemiology studies (3, 10, 11, 13, 31, 32). Our study is consistent with some previous studies (8, 13–15), emphasized that those with earlier-onset type 2 diabetes might be associated with worse macrovascular outcomes than later-onset type 2 diabetes. In the cross-sectional study from the China National HbA1c Surveillance System, patients with early-onset type 2 diabetes had an increased risk of non-fatal CVD than people with late-onset diabetes after further adjustment for diabetes duration (8). A study including 7,844 newly diagnosed diabetes reported that the hazard of macrovascular complication in early-onset type 2 diabetic patients was twice as high in usual-onset diabetes compared with age-matched control subjects (14). Similarly, a recent meta-analysis including 26 observational studies comprising 1,325,493 individuals confirmed that age at diagnosis of diabetes was inversely associated with risk of macrovascular disease, with each 1-year increase in age at diabetes diagnosis associated with a 3% decreased risk of macrovascular disease (OR 0.97, 95%CI 0.96- 0.98) (15). However, some studies suggested there was a positive (16, 17) or null association (9, 18) between age at diagnosis of diabetes and CVD risk. A cross-sectional study from the 2015 Australian National Diabetes Audit (16) suggested that 1 year increase of age at diagnosis of diabetes was associated with increased risk of macrovascular complications (OR, 1.03, 95%CI 1.02-1.04). A 7-years prospective analysis concluded that the higher risk of CVD in patients with young-onset diabetes was driven by longer diabetes duration (9). Results from the Da Qing Diabetes Study indicated that the risk of CVD events did not differ significantly between those age <45 years with diabetes and those age ≥45 years with normal glucose tolerance (HR 1.25, 95% CI 0.93-1.69) (18). The inconsistent findings might be partially explained by the wide variety in population characteristics and the study design. Our study provided evidence from the nation-wide community-dwelling Chinese population, emphasized that those with early-onset diabetes might be associated with worse macrovascular outcomes than late-onset diabetes.

More importantly, we contribute new knowledge about the modifying effect of BMI on the association between early-onset diabetes and CVD risk. Obesity was emphasized to be an important risk factor of diabetes and cardiometabolic disease (33). Over the past four decades, the prevalence of obesity in China has increased steadily, especially in young people. The emerging prevalence of obesity in childhood and adolescent is associated with emergence of comorbidities previously considered to be “elderly” diseases including type 2 diabetes mellitus (34, 35). It was reported that obesity was a more general feature of early-onset type 2 diabetes than it is in later-onset type 2 diabetes (3, 36). A prospective study including 1,462,362 adolescents confirmed that severe obesity significantly increased the risk for incidence of type 2 diabetes in young (20). Importantly, we found that the association of age at diagnosis of diabetes with risk of CVD was more pronounced among patients with obesity or overweight than those with normal weight. Thus, obesity may play an important role in the development of the young-onset phenotype. Interventions of obesity may be effective for the early prevention of CVD among people with early-onset type 2 diabetes.

Potential pathogenetic mechanisms leading to young-onset type 2 diabetes as an aggressive disease compared to late-onset diabetes including the rapid onset of beta cell failure and insulin resistance, and the obesity-related mechanisms (3, 36). Prevailing evidence suggests that young-onset type 2 diabetes has an accelerated decline of beta cell function, especially in second-phase insulin secretion (37, 38). Loss of glycemic control and other metabolic profiles also play essential roles in excessive risk of macrovascular or microvascular complications (5). Recent analysis from Hong Kong found that individuals with early-onset type 2 diabetes had poorly controlled hyperglycemia throughout their life *versus* usual-onset type 2 diabetes (32). Results from the Treatment Options for type 2 Diabetes in Adolescents and Youth Study confirmed that young adults with type 2 diabetes had poor glycemic control regardless of health care coverage (6). Similarly, in our study, those with earlier-onset diabetes tend to have higher HbA1c and triglycerides than patients with later-onset diabetes. Although patients with early-onset diabetes receive more glucose-lowering treatment, they have poor glycemic control. Thus, it is important to improve the effectiveness of blood glucose management for individuals with early-onset diabetes. Besides, younger-onset diabetes often usually has a longer diabetes duration, which was a noticeable traditional risk factor for diabetes complications (16, 17, 39–41).

The study has several strengths. First, the study population was from the REACTION study, which was a multicenter population-based cohort study representing middle-aged and elderly population from various geographical regions in mainland China. Besides, we have comprehensive physical and biochemical measurements, and detailed lifestyle information for the confounding adjustment. However, several potential limitations should be discussed. First, in the current study, the collection of CHD, stroke, and myocardial infarction status was based on self-reported questionnaire. Although we did a validation study of CVD in one of the communities and the validation rate is about 91.1% (29, 30), we still can’t exclude recall bias. Second, the cross-sectional nature may underestimate the risk of CVD associated with early-onset diabetes. Since patients with earlier-onset type 2 diabetes would be more vulnerable to premature death (42). Long-term follow-up study could provide more information in the future. In addition, our study population is limited to 40 years or older. Thus, patients with early-onset diabetes with a relatively short duration were not included in the study.

In conclusion, this large population-based cohort study indicates that age at diagnosis of type 2 diabetes is related to the risk of CVD in patients with type 2 diabetes in China, and this association is more prominent among patients with obesity. The excess risk related to early-onset diabetes mandates more attention to preventive strategies and management guidelines in the Chinese population, and underscore the importance for weight management among early-onset type 2 diabetes.

## Data Availability Statement

The original contributions presented in the study are included in the article/**Supplementary Material**. Further inquiries can be directed to the corresponding authors.

## Ethics Statement

The studies involving human participants were reviewed and approved by the ethic committee of Ruijin hospital Affiliated to Shanghai Jiaotong University School of Medicine. The patients/participants provided their written informed consent to participate in this study.

## Author Contributions

CH, LL, JL, YC, WW, and GN contributed to the concept and design. CH and LL contributed to the acquisition and analysis of data. CH drafted the manuscript. LL, JL and DL edited the manuscript. YJZ, YZ, SYW, JZ, HQ, ML, YYZ, YH, QW, YQ, RH, LS, QS, XY, LY, GQ, XT, GC, MX, YX, TW, ZZ, ZG, GW, FS, ZL, LC, QL, ZY, YFZ, CL, YW, SLW, TY, HD, LLC, TZ, JJZ, YM, YB, WW, GN, YC and JL collected data. All authors made important contributions to critically revising the manuscript for important intellectual content. GN, WW, YB, YC and JL guarantee this work and have full access to the data and take responsibility for the integrity of the data. All authors contributed to the article and approved the submitted version.

## Funding

This study was supported by the Ministry of Science and Technology of the People’s Republic of China (Grant No. 2016YFC1305202, 2017YFC1310700, 2018YFC1311800, 2018YFC1311801), the National Natural Science Foundation of China (Grant No. 81970691, 81970728, 81800683), Karamay Science And Technology Innovation Talent Project (Grant No. 2019RC001A-05), Shanghai Medical and Health Development Foundation (Grant No. DMRFP_I_01), and Shanghai Outstanding Academic Leaders Plan (Grant No. 20XD1422800).

## Conflict of Interest

The authors declare that the research was conducted in the absence of any commercial or financial relationships that could be construed as a potential conflict of interest.

## Publisher’s Note

All claims expressed in this article are solely those of the authors and do not necessarily represent those of their affiliated organizations, or those of the publisher, the editors and the reviewers. Any product that may be evaluated in this article, or claim that may be made by its manufacturer, is not guaranteed or endorsed by the publisher.
