# Supplementary material for: Association Between Age at Diagnosis of Type 2 Diabetes and Cardiovascular Diseases: A Nationwide, Population-Based, Cohort Study
Source: Front Endocrinol (Lausanne). 2021 Oct 4;12:717069. doi: 10.3389/fendo.2021.717069 (PMC8522833; doi:10.3389/fendo.2021.717069)
Supplement: Supplementary file 1 [file Table_1.docx]

**Supplementary Table 1. Association between Age at Diagnosis of Diabetes and Cardiovascular Disease Risk by gender.**

|  | **Cardiovascular disease** | | **Stroke** | | **Myocardial infarction** | | **Coronary heart disease** | |
| --- | --- | --- | --- | --- | --- | --- | --- | --- |
|  | **Case (%)** | **OR (95%CI)** | **Case (%)** | **OR (95%CI)** | **Case (%)** | **OR (95%CI)** | **Case (%)** | **OR (95%CI)** |
| **Men** |  |  |  |  |  |  |  |  |
| ≥60 | 500 (19.28) | 1.00 (reference) | 107 (4.78) | 1.00 (reference) | 65 (2.51) | 1.00 (reference) | 354 (13.65) | 1.00 (reference) |
| 50-59 | 549 (14.39) | 1.41 (1.19-1.68) | 107 (3.08) | 1.32 (0.99-1.77) | 84 (2.20) | 1.31 (0.87-1.96) | 386 (10.12) | 1.42 (1.17-1.73) |
| 40-49 | 275 (10.37) | 1.60 (1.28-2.00) | 47 (2.11) | 1.63 (1.12-2.38) | 59 (2.22) | 1.52 (0.92-2.53) | 186 (7.01) | 1.57 (1.20-2.03) |
| 18-39 | 64 (9.57) | 1.78 (1.23-2.57) | 10 (1.75) | 1.58 (0.83-3.01) | 10 (1.49) | 1.29 (0.57-2.94) | 47 (7.03) | 1.88 (1.23-2.88) |
| Per 5-years earlier |  | 1.15 (1.10-1.21) |  | 1.12 (1.04-1.20) |  | 1.11 (1.01-1.23) |  | 1.15 (1.09-1.21) |
| **Women** |  |  |  |  |  |  |  |  |
| ≥60 | 837 (20.80) | 1.00 (reference) | 163 (5.54) | 1.00 (reference) | 59 (1.47) | 1.00 (reference) | 667 (16.57) | 1.00 (reference) |
| 50-59 | 926 (15.40) | 1.28 (1.12-1.46) | 167 (3.88) | 1.23 (0.96-1.57) | 53 (0.88) | 1.16 (0.73-1.84) | 756 (12.57) | 1.31 (1.13-1.52) |
| 40-49 | 417 (12.09) | 1.47 (1.22-1.77) | 86 (3.19) | 1.35 (0.96-1.89) | 25 (0.72) | 1.41 (0.76-2.61) | 339 (9.83) | 1.46 (1.19-1.79) |
| 18-39 | 85 (11.42) | 1.70 (1.26-2.30) | 16 (2.86) | 1.63 (0.93-2.86) | 10 (1.34) | 3.90 (1.77-8.60) | 66 (8.87) | 1.55 (1.12-2.17) |
| Per 5-years earlier |  | 1.14 (1.09-1.18) |  | 1.10 (1.03-1.18) |  | 1.23 (1.10-1.38) |  | 1.13 (1.08-1.18) |

Adjusted for age, education, smoking status, drinking status, physical activity, healthy dietary, body mass index, systolic blood pressure, total cholesterol, triglycerides and HbA1c. P for interaction for cardiovascular disease = 0.4379; p for interaction for stroke = 0.8955; p for interaction for myocardial infarction= 0.5334; p for interaction for coronary heart disease = 0.2824.

**Supplementary Table 2. Odds Ratio (95% CI) for Cardiovascular Disease Risks According to Age at Diagnosis of Diabetes.**

|  | **Age at diagnosis of diabetes (years)** | | | | |
| --- | --- | --- | --- | --- | --- |
|  | **≥60** | **50-59** | **40-49** | **18-39** | **Per 5-years earlier** |
| **Cardiovascular disease, case (%)** | 1337 (20.20) | 1475 (15.01) | 692 (11.34) | 149 (10.54) |  |
| Model 1 | 1.00 (reference) | 1.25 (1.09-1.79) | 1.32 (1.13-1.54) | 1.39 (1.09-1.79) | 1.10 (1.07-1.14) |
| Model 2 | 1.00 (reference) | 1.24 (1.11-1.38) | 1.30 (1.12-1.50) | 1.38 (1.08-1.75) | 1.10 (1.06-1.13) |
| Mode 3 | 1.00 (reference) | 1.24 (1.11-1.38) | 1.30 (1.12-1.50) | 1.37 (1.08-1.75) | 1.10 (1.06-1.13) |
| **Stroke, case (%)** | 350 (5.29) | 365 (3.71) | 174 (2.85) | 37 (2.62) |  |
| Model 1 | 1.00 (reference) | 1.19 (0.99-1.44) | 1.31 (1.01-1.69) | 1.36 (0.88-2.10) | 1.08 (1.02-1.14) |
| Model 2 | 1.00 (reference) | 1.20 (0.99-1.45) | 1.31 (1.01-1.70) | 1.39 (0.90-2.14) | 1.08 (1.02-1.14) |
| Model 3 | 1.00 (reference) | 1.20 (0.995-1.45) | 1.31 (1.01-1.70) | 1.39 (0.90-2.14) | 1.08 (1.02-1.14) |
| **Myocardial infarction, case (%)** | 124 (1.87) | 137 (1.39) | 84 (1.38) | 20 (1.42) |  |
| Model 1 | 1.00 (reference) | 1.17 (0.86-1.59) | 1.34 (0.90-2.00) | 1.80 (0.999-3.23) | 1.14 (1.05-1.23) |
| Model 2 | 1.00 (reference) | 1.20 (0.88-1.63) | 1.37 (0.92-2.04) | 1.90 (1.05-3.44) | 1.14 (1.06-1.24) |
| Model 3 | 1.00 (reference) | 1.20 (0.88-1.63) | 1.37 (0.92-2.04) | 1.89 (1.05-3.41) | 1.14 (1.06-1.24) |
| **Coronary heart disease, case (%)** | 1021 (15.43) | 1142 (11.62) | 525 (8.60) | 113 (8.00) |  |
| Model 1 | 1.00 (reference) | 1.24 (1.10-1.40) | 1.25 (1.06-1.48) | 1.28 (0.98-1.68) | 1.08 (1.05-1.12) |
| Model 2 | 1.00 (reference) | 1.25 (1.10-1.41) | 1.25 (1.06-1.48) | 1.29 (0.98-1.69) | 1.08 (1.05-1.12) |
| Model 3 | 1.00 (reference) | 1.25 (1.11-1.41) | 1.25 (1.06-1.48) | 1.29 (0.98-1.69) | 1.08 (1.05-1.12) |

Model 1: adjusted for age, sex, education, smoking status, drinking status, physical activity, healthy dietary, body mass index, systolic blood pressure, total cholesterol, triglycerides, HbA1c, insulin therapy and oral hypoglycemic drugs uses; Model 2: adjusted for age, sex, education, smoking status, drinking status, physical activity, healthy dietary, body mass index, systolic blood pressure, total cholesterol, triglycerides, HbA1c, insulin therapy, oral hypoglycemic drugs uses, lipid-lowering treatment and antihypertensive drugs use; Model 3: Adjusted for model 2 plus probable depression.
